# Lithium as a disease-modifying agent for prion diseases

**DOI:** 10.1038/s41398-018-0209-4

**Published:** 2018-08-22

**Authors:** A. Relaño-Ginés, S. Lehmann, E. Brillaud, M. Belondrade, D. Casanova, C. Hamela, C. Vincent, S. Poupeau, J. Sarniguet, T. Alvarez, J. D. Arnaud, J. C. Maurel, C. Crozet

**Affiliations:** 1 Institut de Médecine Régénératrice et de Biothérapie (I.M.R.B.), Physiopathologie, diagnostic et thérapie cellulaire des affections neurodégénératives—Institut National de la Santé et de la Recherche Médicale Université de Montpellier U1183 Centre Hospitalo, Universitaire de Montpellier, Montpellier, France; 2 Institut de Génétique Humaine, Centre National de la Recherche Scientifique-UPR1142, Montpellier, France; 3 Medesis Pharma SA, Avenue du Golf, Baillargues, France; 4 Etablissement Confiné d’Expérimentation BioCampus, Université Montpellier, Campus Triolet, Bâtiment 53, CECEMA, Montpellier, France

## Abstract

Prion diseases still remain incurable despite multiple efforts to develop a treatment. Therefore, it is important to find strategies to at least reduce the symptoms. Lithium has been considered as a neuroprotective agent for years, and the objective of this preclinical study was to evaluate the efficacy of lithium delivered as a water-in-oil microemulsion (Aonys^®^). This delivery system allows using low doses of lithium and to avoid the toxicity observed in chronic treatments. C57BL/6J mice were intracranially inoculated with ME7 prion-infected brain homogenates and then were treated with lithium from day 90 post inoculation until their death. Lithium was administered at traditional doses (16 mg/kg/day) by the gavage route and at lower doses (40 or 160 µg/kg/day; Aonys^®^) by the rectal mucosa route. Low doses of lithium (Aonys^®^) improved the survival of prion-inoculated mice, and also decreased vacuolization, astrogliosis, and neuronal loss compared with controls (vehicle alone). The extent of the protective effects in mice treated with low-dose lithium was comparable or even higher than what was observed in mice that received lithium at the traditional dose. These results indicate that lithium administered using this innovative delivery system could represent a potential therapeutic approach not only for prion diseases but also for other neurodegenerative diseases.

## Introduction

Prion diseases are progressive neurodegenerative disorders caused by conversion of a normal cell-surface glycoprotein (PrP^C^) into a conformationally altered isoform (PrP^Sc^) that is infectious. This abnormal PrP^Sc^ is a protease-resistant isoform of the host-encoded PrP^C^. Conformational differences between PrP^C^ and PrP^Sc^ are evidenced by an increase of β-sheet content as well as by the protease resistance of the PrP^Sc^. Prion diseases are characterized by rapid neuronal cell death, vacuolization, and gliosis. Prion diseases affect humans and animals, and, today, they still remain incurable. Patients with prion diseases suffer from cognitive deficits, movement problems, ataxia, as well as swallowing, visual, communication, and mood disturbances. Different therapeutic strategies have been tested in preclinical prion models with limited results; therefore, it is important to find approaches for palliating at least the disease symptoms.

A growing body of evidence suggests that lithium may have neuroprotective effects^1–7^. Lithium is beneficial for bipolar disorders and dementia^8^. Different studies have reported that lithium can modify the pathology in some neurodegenerative disorders, such as Alzheimer’s disease (AD)^2^, and Huntington’s disease (HD)^3,4,9–11^. Moreover, lithium can reduce PrP^Sc^ aggregates in cultured prion-infected neuronal and non-neuronal cells by mTOR-independent autophagy induction^1,12,13^. Specifically, lithium promotes not only PrP^Sc^ degradation, but also limits the amount of PrP^C^ available for conversion into PrP^Sc^ through autophagy induction^13^. Therefore, lithium is considered to be an autophagy inducer, leading to upregulation of the autophagy pathway^1^, and its neuroprotective effects in neurodegenerative diseases might be partly explained by this feature.

These studies suggest that lithium could be beneficial for the treatment of neurodegenerative diseases; however, several works have also reported the presence of irreversible lithium-induced toxicity^14,15^. In its conventional formulation, lithium has a narrow therapeutic window with safe plasma concentrations between 0.5 and 1.2 mEq/L in humans^16^. Toxic effects usually occur at 1.5 mEq/L and dangerous life-threatening side effects are observed at concentrations above 2.0 mEq/L^11,16,17^. This narrow therapeutic range complicates the use of lithium for long-term treatments^18,19^.

Interestingly, a new vector, named Aonys^®^, has been recently developed to deliver various molecules^20,21^. This novel delivery technology allows the delivery and large body distribution of significantly lower doses of a drug of interest without compromising its pharmacological effects. This property seems to be due to the water-in-oil microemulsion of the Aonys^®^ vector that allows the drug diffusion through mucosae. The Aonys^®^ vector has been already successfully used by our laboratory to deliver anti-PrP siRNAs^22^ with a reduction of the pathological lesions in a prion disorder mouse model. The same vector system has also been used to produce NP03, a lithium microemulsion in which lithium citrate is mixed with Aonys^®^^23^. This formulation offers the possibility to lower by several hundred times the quantity of lithium to be used compared with conventional formulations. NP03 has already been tested in the YAC128 mouse model of HD^11^ and in a well-characterized rat model of AD^24^. In the YAC128 mouse model, NP03 ameliorated the motor and neuropathological phenotypes and improved various biochemical features^11^. In the rat model of AD, NP03 reversed the deficits in novel object recognition, inactivated GSK-3β through phosphorylation at Ser9, rescued the native β-catenin protein levels, and decreased *BACE1* gene expression. NP03 also reduced the levels of toxic Aβ42 peptides and induced neurogenesis, as indicated by the increase in doublecortin expression in hippocampus tissue sections^24^. Based on these encouraging results, we wanted to determine whether treatment with low doses of lithium (using NP03) could improve prion-related pathology in a commonly used model of prion disease obtained by inoculating ME7 prion-infected brain homogenates in the brain of C57BL/6J mice. This prion mouse model is relevant to mimic prion disorders and the mouse-adapted strain ME7 is, with RML 139A and 79A, one of the most murine-adapted prion strain used for preclinical studies^25^. To this aim, we initiated lithium-based treatment at day 90 post inoculation and continued until the animals’ death (between 165 and 175 days post inoculation). Mice received lithium either at a high dose (16 mg/kg/day) by the gavage route or at lower doses (40 or 160 µg/kg/day; Anoys^®^ NP03 lithium microemulsion) by the rectal-per-mucosal route because NP03 can only be absorbed by mucosal administration. The rectal route was selected because repeated buccal-per-mucosal administration in the mice is more difficult and requires anesthesia that could influence the study results.

Our results demonstrate that low doses of lithium delivered by NP03 not only improved the survival of prion-inoculated mice, but also decreased vacuolization, astrogliosis, and neuronal loss. This suggests that lithium delivery by this new system could represent a potential valuable therapeutic approach for human prion diseases.

## Materials and methods

### Animal model

This study was approved by the appropriate ethical committee “Comité régional Languedoc-Roussillon d’éthique en matière d’expérimentation animale” (UFR de Pharmacie, Avenue Charles Flahault 34060, Montpellier, France) under the number CEEA-LR-1019. Mice were housed in an A3 facility according to the European Community Directive 86/609/EEC with ad libitum access to food and water. In total, 55 C57BL/6J female mice (from Charles Rivers Laboratory) were used for this study. All animals were acclimated at least 5 days to animal facilities before inclusion in the study. Each cage will contain 4–5 animals that have been randomly separated in different cages upon their arrival. Brain inoculation of the ME7 prion strain (1%, 20 µl) in 5-week-old mice was performed using a Myjector Insulin Syringe (Terumo) and under general anesthesia (100 µg/g ketamine from Imalgène + 15 µg/g xylazine from Rompun). Mice were observed daily for the appearance of prion-related clinical signs and were finally sacrificed for ethical reasons following anesthesia and cervical dislocation.

### Preparation of Aonys^®^/lithium vector

Water-in-oil microemulsion containing either water alone (vehicle) or lithium citrate salts (Li_3_C_6_H_5_O_7_, 4H_2_O; NP03) was provided by MedesisPharma.

Lithium was dissolved in nuclease-free water (Qiagen) and stored at −20 °C until use. Each day, the quantity necessary to treat the animals was sampled from the vial after mixing. According to a well-defined ratio, NP03 is formed rapidly upon short vortexing of the Aonys^®^ lipid mixture and lithium solution^23^.

### Treatment delivery

At day 90 after prion inoculation, mice were separated into four groups randomly (Table 1). Sample size was estimated based on our previous work^22^. The control group (*n* = 13) received NP03/vehicle; the NP03-40 group (*n* = 14) received NP03-lithium at a dose of 40 µg/kg per day; the NP03-160 group (*n* = 14) received NP03-lithium at a dose of 160 µg/kg per day (all by the rectal route); and the lithium-gavage group (*n* = 14) received lithium solution at a dose of 16 mg/kg per day (the classical lithium dose used in mice) by gavage. All mice received their treatment five times per week. The investigators were not blinded to the group allocation. A constant dosage-volume of 1 ml/kg was used for mucosal (by rectal) administration using a micropipette and adapted conical tips. The possible rejection was documented and the quantity of lithium solution administered to each animal was adjusted according to the mean of the most recently recorded body weights and a constant dosage-volume of 5 ml/kg was used.

**Table 1:** Summary of the control and treatment groups used in the study

Treatment	Number of mice	Route	Lithium dose (µg/kg/day)
Vehicle (control)	13	Rectal	0
NP03-lithium	14	Rectal	40
NP03-lithium	14	Rectal	160
Lithium solution	14	Oral	16,000

### Tissue sampling

At death, brains were removed and cut into two parts: the first half was fixed in 4% paraformaldehyde for immunohistological analyses, and the other half was frozen at −80 °C for biochemical analyses.

### Histological analysis

Brains were manually embedded in paraffin. Brain sections of 5 µm were cut using a Microm microtome and collected on Strafrost glasses (Microm, France). Tissues were dewaxed using the Clearify solution (American Master Tech Scientific, Inc.) and rehydrated through a decreasing gradient of ethanol washes. For histological studies, samples were stained with hematoxylin (Gill’s formula H-3401, Vector Laboratories) at room temperature for 3 min, washed with water, and treated with acid ethanol. They were then washed with water and incubated with 2% eosin at room temperature for 3 min and washed again.

#### Glial fibrillary acidic protein (GFAP), NeuN, and LC3 immunohistochemistry

Sections were pre-incubated with 20 mg/ml proteinase K (Roche) at 37 °C for 10 min for GFAP (Dako) immunostaining, with 1 mM EDTA buffer for 20 min in a microwave for NeuN (Millipore) immunostaining, and with Tris-EDTA buffer for LC3 (Sigma-Aldrich) immunostaining. They were then washed with water and immersed in 0.5% hydrogen peroxide at room temperature for 20 min to inhibit endogenous peroxidases. After saturation in PBS/0.1% Triton X-100/0.1% BSA for 1 h, sections were incubated at 4 °C with anti-GFAP (1:100), anti-NeuN (1:600), or anti-LC3 (1:600) primary antibodies (in PBS/0.1% Triton X-100/0.1% BSA) overnight. After incubation with the secondary biotinylated goat anti-rabbit (Amersham) or biotinylated goat anti-mouse antibodies (Vector Laboratories) (1:1000 in PBS/0.1% Triton X-100), the avidin–peroxidase complex (Vectastain Elite kit, Vector Laboratories) was added, followed by 3,3′-diaminobenzidine (DAB) (Vector Laboratories), according to the manufacturers’ instructions.

#### PrP^Sc^ immunohistochemistry

PrP^Sc^ was revealed using the SAF84 anti-PrP monoclonal antibody (0.5 mg/ml) that recognizes the 161–170 sequence of human PrP (kindly provided by Dr. J. Grassi, CEA/SPI, Saclay, France). For epitope retrieval, tissue sections were incubated in formic acid (10 min) followed by autoclaving at 121 °C for 10 min. The secondary antibody was a biotinylated goat anti-mouse antibody (Amersham, France) (1:1000 in PBS/0.1% Triton X-100). The avidin–peroxidase complex (Vectastain Elite kit, Vector Laboratories, Clinisciences) was then added, followed by DAB.

Slides were scanned using a Nanozoomer Slide Scanner (Hamamatsu, platform MRI, INM Montpellier). The number (number/mm^2^) of vacuoles or of LC3-II-positive cells and the surface area (µm^2^/mm^2^) of astrocytes and neurons were determined using the ImageJ software.

### Western blot analysis

Brain tissues were homogenized in PBS to obtain a 10% brain homogenate (w/v). After proteinase K digestion (200 µg/100 mg of tissue), brain samples were loaded onto 12% SDS/PAGE Nupage gels (Invitrogen). Immunoblotting was performed using standard procedures as previously reported^22,26^. PrP^Sc^ was detected using a mixture of three monoclonal antibodies (SAF60, SAF69, and SAF70), called SAFmix, and revealed by chemiluminescence (Pierce). Semi-quantification of the signal was performed with the XRS Bio-RAD CDD Camera imaging system. For PrP^C^ western blot analysis, samples were not submitted to PK digestion and we used the monoclonal SAF32 anti-PrP antibody that recognizes the N-terminal part of PrP^C^.

### Statistical analysis

Data are expressed as the mean ± S.E.M. We used GraphPad Prism 7 to check for variance and normality. This led us to interpret the results with Mann–Whitney non-parametric tests. The significance level was defined at 0.05 and *p < 0.05, **p < 0.01, and ***p < 0.001 in all figures. Statistical analyses were performed with GraphPad Prism 7 (GraphPad Software).

## Results

### Lithium improves survival in prion-infected mice

After intracranial inoculation of ME7 prion-infected brain homogenates, C57BL/6J mice were separated in four treatment groups: control mice (Aonys^®^ vehicle alone; *n* = 13), NP03-40 and NP03-160 groups (*n* = 14/each; 40 or 160 µg of lithium/kg, respectively), and lithium-gavage group (*n* = 14; 16 mg of lithium/kg by gavage) (Table 1). All treatments (lithium or vehicle) were initiated at day 90 when cognitive impairment begins for this model and continued until the death of animals which typically occurs at day 170 ± 5 for the untreated mice.

**Fig. 1:**
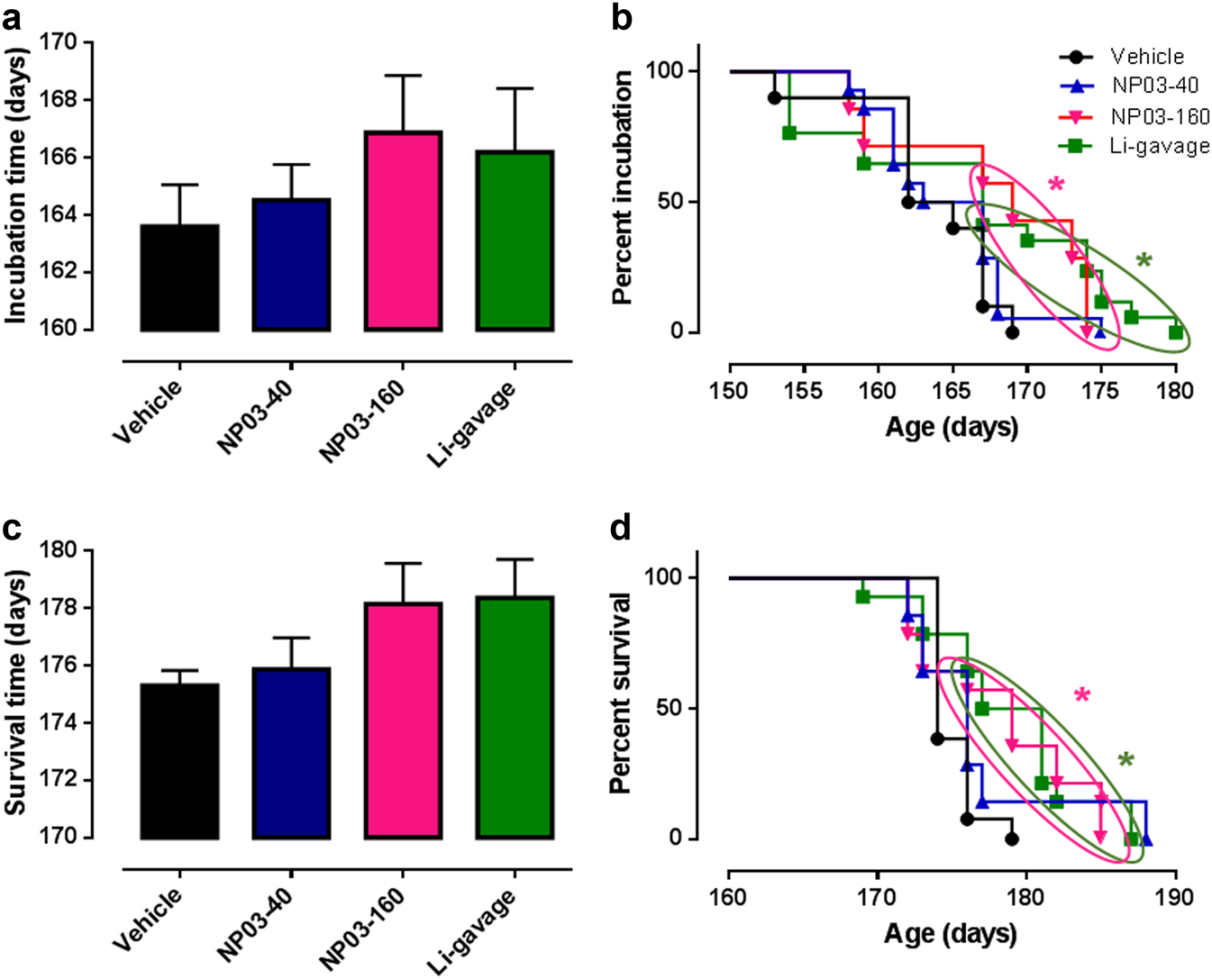
Incubation and survival time of control mice (vehicle; *n* = 10), mice treated with NP03-40 (*n* = 14), NP03-160 (*n* = 14), and lithium by gavage (*n* = 14) from day 90 after intracranial inoculation of ME7 prion-infected brain homogenates. **a** Incubation time (mean ± SEM). No significant difference was found with the Mann–Whitney test. **b** Kaplan–Meier curves showing the percentage of incubation time per mouse for each group. **c** Survival time (mean ± SEM); the unpaired Mann–Whitney test was used for statistical analysis. **d** Kaplan–Meier survival curve showing the percent of survival for each mouse per group

Although the total incubation period in this model is approximately 24 weeks, PrP^Sc^ and glial cell responses such as gliosis can be detected shortly after inoculation. This is followed by synaptic loss approximately at 12 weeks post inoculation, neuronal loss 15 days later, and the clinical onset of the disease can finally be observed at approximately 20 weeks^27^. The incubation time, which was determined by the appearance of at least three clinical signs (including waddling gait, flattened back, rough coat, sticky eye discharge, weight loss, very jumpy, hunched, or incontinence), was not significantly different in the four groups (Fig. 1a), although it was slightly longer in the three lithium-treated groups. However, mice in the same group did not respond to treatment in the same manner, as it is shown by the Kaplan–Meier incubation curves, where only some mice (indicated within the colored circles) exhibited longer incubation times (Lithium-gavage or NP03-160) in a statistically significant way compared to mice in the vehicle control group (vehicle *n* = 5; gavage: *n* = 8/*p* = 0.0391; NP03-160: *n* = 9/*p* = 0.011; Fig. 1b).

Similarly, survival time, which was the disease endpoint, was longer in the three groups treated with lithium compared with the control group, but this difference was not significant (Fig. 1c). However, the mice in the lithium-gavage and NP03-160 groups that died after the mean survival time of the control group (colored circles in Fig. 1d) presented a survival time significantly longer than the vehicle control group (control *n* = 5; lithium-gavage: *n* = 11, *p* = 0.0229; NP03-160: *n* = 9, *p* = 0.0170). Interestingly, the low dose of 160 µg/kg delivered through the NP03 formulation showed the same effect as that observed when using a much higher dose (16 mg/kg per day) by conventional gavage method. If we consider the mice treated with the vehicle that died after the mean incubation time, there is a slight increase between 2 and 7.5% of the incubation and survival time.

### Effect on PrP^Sc^ accumulation

**Fig. 2:**
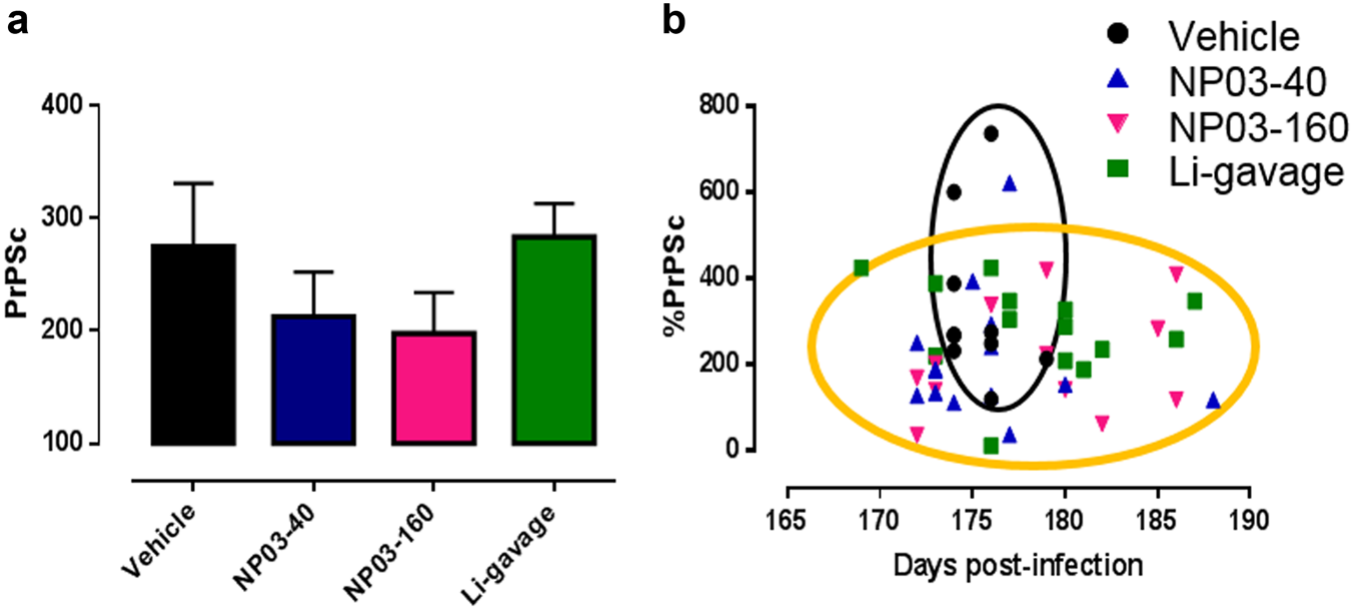
Postmortem analysis of PrP^Sc^ accumulation in control mice (vehicle; *n* = 10), and mice treated with NP03-40 (*n* = 14), NP03-160 (*n* = 14), and lithium by gavage (*n* = 14). **a** Quantification (mean ± SEM) of the PrP signal after immunoblotting of brain protein extracts with the SAFmix of anti-PrP antibodies; data were normalized to the reference signal of a ME7 brain homogenate pool. **b** Individual PrP^Sc^ levels for each mouse as a function of its survival time

Accumulation of PrP^Sc^ aggregates is one of the main hallmarks of prion disease, and typically appears 8 weeks after prion inoculation in the model used for this study. At death, half of each brain was used for PrP^Sc^ detection by western blot analysis after proteinase K digestion. PrP^Sc^ accumulation was not significantly different (Mann–Whitney test) in the four groups, although lower PrP^Sc^ levels were observed in the NP03-40 and NP03-160 groups (Fig. 2a).

Moreover, no significant correlation was found between the individual PrP^Sc^ levels and survival time (Fig. 2b). However, vehicle-treated mice were mainly grouped in a narrow range of days post infection and showed the highest PrP^Sc^ levels, whereas in lithium-treated groups, PrP^Sc^ levels were more dispersed in time and do not reach the high level of PrP^Sc^ as in the control mice (Fig. 2b).

PrP^C^ brain levels were also analyzed in controls and lithium-treated mice by western blot. We did not observe any difference in PrP^C^ signal intensity between controls (vehicle) and the NP03-40 and NP03-160 groups, as shown in Supplementary Fig. 1. PrP^C^ was not affected by the treatment excluding that lithium efficacy is linked to a modulation of PrP^C^ expression (Supplementary Fig. 1).

### Effect of NP03 treatment on vacuolization, astrocytosis, neuronal count, and autophagy

**Fig. 3:**
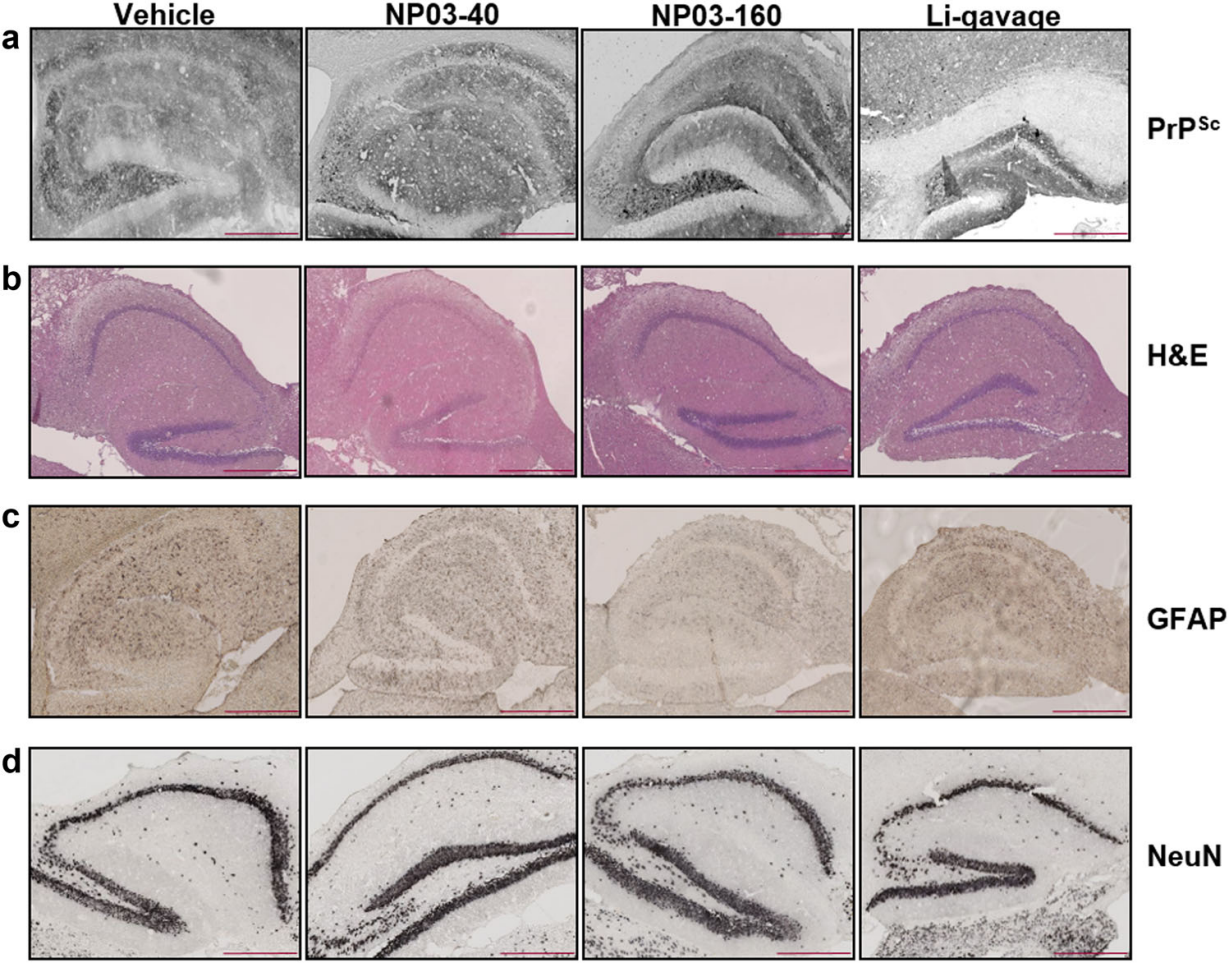
**a** Representative images of the postmortem immunohistological analysis of hippocampus tissue sections to evaluate **a** PrP^Sc^ accumulation using the SAF84 antibody, **b** the presence of vacuoles (arrows) after hematoxylin–eosin staining, **c** GFAP-positive astrocytes, and **d** NeuN (neuronal marker)-positive cells. Scale bar 400 µm

Immunostaining of 5-µm paraffin-embedded brain tissue sections using the anti-PrP^Sc^ SAF84 antibody (Fig. 3a) showed the typical PrP^Sc^ immunostaining patterns (diffuse, ponctiform, and amyloid deposits) in each brain with no difference among groups, in agreement with the western blot results. Histological analysis was done to evaluate vacuolization of the neuropil (Fig. 3b), and immunohistological analysis of astrocytes (GFAP) and neurons (NeuN) was performed to study astrocytosis and the variation of neuronal numbers, respectively (Fig. 3c, d).

#### Vacuolization

**Fig. 4:**
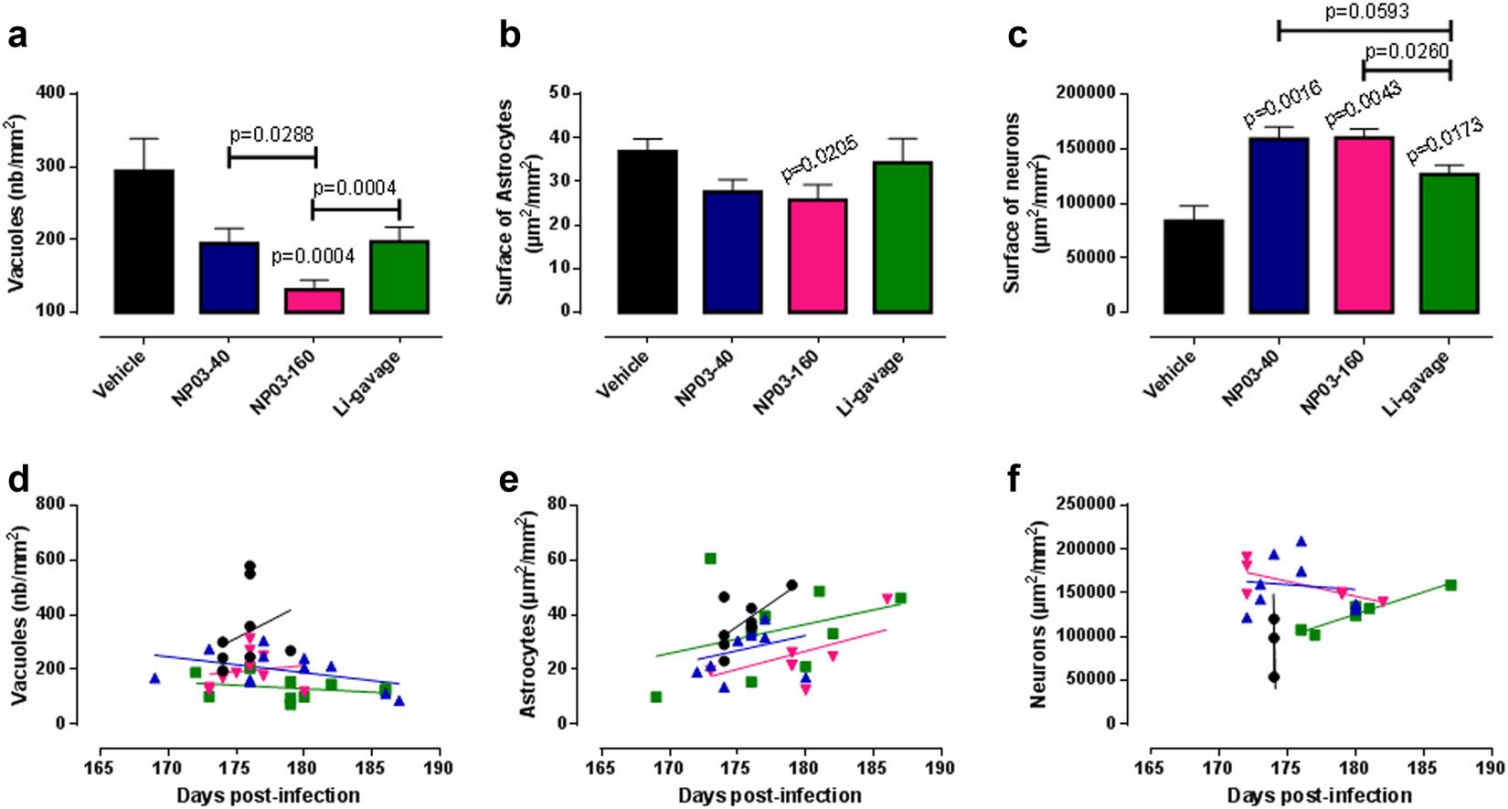
Quantification (mean ± SEM) of the number of vacuoles/mm^2^ in hippocampus sections after hematoxylin–eosin staining (*n* = 11 control; *n* = 11 NP03-40; *n* = 10 NP03-160; *n* = 10 lithium-gavage). **a** Quantification of the surface of GFAP-positive astrocytes (*n* = 10 control; *n* = 10 NP03-40; *n* = 8 NP03-160; *n* = 9 lithium-gavage) and **b** of the surface occupied by NeuN-positive neurons (*n* = 5 control; *n* = 6 NP03-40; *n* = 7 NP03-160; *n* = 6 lithium-gavage). **c** The unpaired Mann–Whitney test was used for statistical analysis in all cases. **d**–**f** Individual vacuole number and GFAP-positive and NeuN-positive cell surface as a function of the individual survival time

After hematoxylin and eosin staining of 5-µm paraffin-embedded brain tissue sections to detect vacuolar lesions (Fig. 3b), the number of vacuoles (nb/mm^2^) was determined in the hippocampus (Fig. 4a, d). Compared with vehicle control group, vacuole number was significantly lower in the three lithium-treated groups, with the highest reduction in the NP03-160 group (*p* < 0.001 compared with the control group, Mann–Whitney test). The vacuole number reduction in the NP03-160 group was significantly more important compared with the other two lithium-treated groups (NP03-40 and Li-gavage) (Fig. 4a). This result is interesting because the highest effect was found when using one of the lowest doses of lithium (NP03-160). In fact, the results obtained from mice treated with the highest dose of lithium by gavage presented the same effect found in the mice treated with the lowest dose of lithium using the NP03 vector but they are higher (*p* < 0.05) than in NP03-160-treated groups (Fig. 4a). The analysis of the correlation between the individual survival times and vacuole numbers showed that for the lithium groups, there is more dispersion over time and there are no high values for vacuole numbers, contrary to what happens in the control group (Fig. 4d). This is similar to the results obtained for the individual PrP^Sc^ data (Fig. 2b).

#### Astrocytosis

To determine whether astrogliosis, a hallmark of prion disease, was modified by lithium treatment, immunohistochemical analysis of the GFAP astrocyte marker was performed (Fig. 3c). Glial proliferation is typically found in prion disorders^28^. Quantification of the hippocampus surface (µm^2^/mm^2^) covered by GFAP-positive astrocytes showed a significantly lower area only in the NP03-160 group compared with the control group (Fig. 4b). The surface was highly reduced in the NP03-40 group (*p*-value = 0.0753) compared to the vehicle control. No correlation between survival time and astrocyte surface could be observed (Fig. 4e), although in the lithium-treated groups, this surface tended to increase over time.

#### Evaluation of neuronal loss/recovery

Immunohistochemical analysis of the NeuN neuronal marker (Fig. 3d) followed by quantification of the hippocampus surface covered by NeuN-positive neurons (µm^2^/mm^2^) showed that this surface area was significantly larger in the three lithium groups compared with control mice. It was also larger in the NP03-40 and NP03-160 groups compared with the vehicle-treated mice (*p* < 0.005 for NP03-40 and NP03-160; Fig. 4c). In fact, the difference between the number of neurons in the NP03-160 group and mice treated with the highest dose of lithium by gavage was also statistically significant (*p* = 0.0260; Fig. 4c). Interestingly, a positive correlation between neuronal surface and survival time seems to occur for the gavage-treated and NP03-40-treated mice (Fig. 4f), in which the increasing number of neurons are observed at the end of the disease.

#### Autophagy

**Fig. 5:**
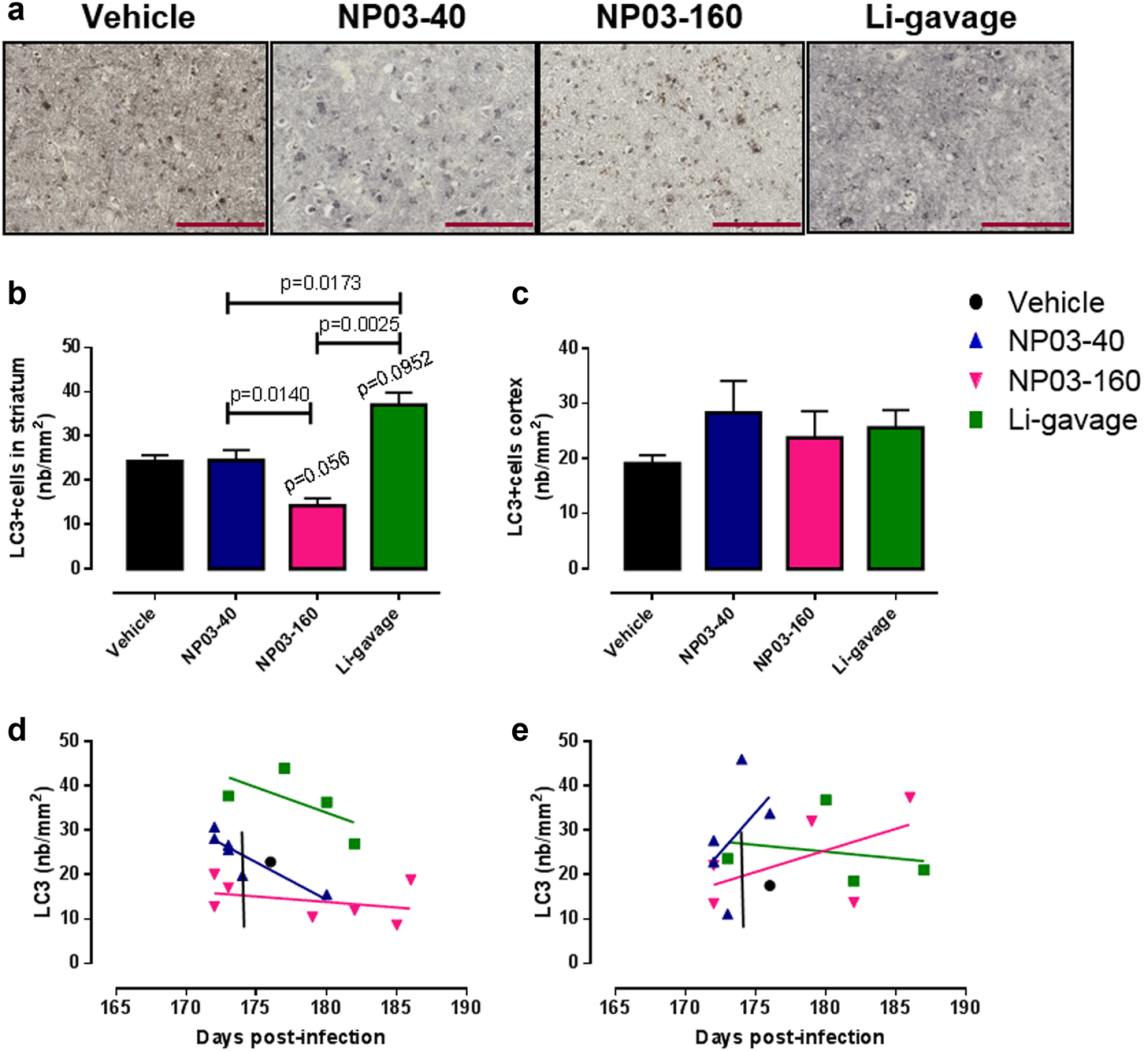
**a** Immunohistological analysis of LC3 expression (representative images). **b**, **c** Quantification of LC3-positive cells (mean ± SEM) in the striatum and cortex, respectively (*n* = 2 control; *n* = 5 NP03-40; *n* = 6 NP03-160; *n* = 7 lithium-gavage, for striatum; and *n* = 2 control; *n* = 5 NP03-40; *n* = 5 NP03-160; *n* = 5 lithium-gavage, for cortex). The unpaired Mann–Whitney test was used for statistical analysis in all cases. **d**, **e** Individual LC3 levels as a function of the survival time. Scale bar 400 µm

Autophagy activation leads to an increase of LC3-II associated with the autophagosomal membrane, and thus to a punctate LC3-II staining. The increase in LC3-II expression is currently used as a marker of autophagy induction^29^. Immunohistological analysis of LC3-II expression (Fig. 5a) and quantification of the number of LC3-II-positive cells in the striatum (nb/mm^2^) (Fig. 5b) showed that compared with the vehicle group, the number of LC3-II-positive cells was significantly increased only in the lithium-gavage group, whereas it was decreased in the NP03-160 group (*p* = 0.056, Fig. 5b). No effect was instead observed in the mice treated with the lowest dose of lithium (NP03-40) (Fig. 5b). Quantification of the number of LC3-II-positive cells in the cortex did not highlight any significant difference between control and treated mice (Fig. 5c). Similarly, no specific correlation between the number of LC3-II-positive cells and survival time was observed (Fig. 5d, e). These data indicate that the effects can be different depending on the analyzed brain region, and suggest that the striatum could be more sensitive to lithium-dependent autophagy changes.

## Discussion

Lithium was first used in 1949, and since then it has become the most commonly used drug for the treatment of bipolar disorders and depression^30–33^. More recently, it was suggested that lithium benefits could be extended beyond mood stabilization. Particularly, lithium has been tested in several neurodegenerative diseases, such as Parkinson’s disease, AD, HD, and amyotrophic lateral sclerosis (ALS), because of its neuroprotective properties^1–7^. Lithium has been considered as a candidate drug for disease modification in neurodegenerative disorders due to its role in the modulation of multiple biological cascades involved in neuroprotective mechanisms, such as the activation of neurotrophic responses, upregulation of mitochondrial function, decrease of the inflammatory response, increase of anti-apoptotic protein expression, increase of hippocampal neurogenesis, and autophagy^34–37^.

Although these studies suggest that lithium may be beneficial for the treatment of neurodegenerative diseases, several works have also reported the presence of irreversible lithium-induced toxicity^14,15^. In its conventional formulation, lithium has a narrow therapeutic window with safe plasma concentrations between 0.5 and 1.2 mEq/L in humans^16^. Toxic effects usually occur at 1.5 mEq/L and dangerous life-threatening side effects can occur at concentrations above 2.0 mEq/L^16,11,17^. Therefore, this narrow therapeutic range complicates the use of lithium for long-term treatments^18,19^.

Recently, some studies highlighted the benefits of Aonys^®^, a novel delivery technology that allows an important body distribution and the delivery of significantly lower doses of a drug of interest without compromising its pharmacological effects. This property seems to be due to the water-in-oil microemulsion of the Aonys^®^ component that allows the drug diffusion through mucosae. Our laboratory has already used this technology to deliver anti-PrP siRNAs for 12 days with a reduction by 28% of PrP^c^ in the brain of prion-infected mice^22^. Aonys^®^ has also been tested in HD mice to deliver low doses of lithium^11^ or peptides^38^, and in AD rats to deliver low doses of lithium^24^. All these studies showed that Aonys^®^ is safe and does not cause adverse effects, and that it could represent an alternative to intravenous or subcutaneous lithium administration. For human treatment, one could envisage the administration of low doses of NP03 lithium through oral mucosae using an oral spray. This would not only represent an easy-to-perform treatment, but also a good way to avoid the overdosing-related toxicity problems, usually observed with traditional lithium doses.

In the present study, we compared the effects in a prion experimental model of a high dose of conventional lithium administered by gavage with low lithium treatments delivered by Aonys^®^ formulation in the form of NP03. In prion diseases, lithium treatment was tested in murine neuroblastoma cells persistently infected with the RML scrapie strain (RML-N2a cells)^13^ and in Syrian hamsters infected with the 263K scrapie strain^39^. Incubation of infected neuroblastoma cells with lithium led not only to a reduction of PrP^Sc^ aggregates, but also to autophagy induction and reduction of the levels of cellular prion protein, thus limiting the substrate available for PrP^Sc^ conversion. In scrapie-infected hamsters, lithium aluminum hydride can destroy PrP^Sc^ and extend the incubation period^39^. In the present study, we started lithium treatment when cognitive impairment, PrP^Sc^ accumulation, neuronal loss, and gliosis were already present^27^, in order to mimic the therapeutic situation. Despite the advanced disease stage at the beginning of treatment, survival was extended in all lithium-treated mice, even with the lowest dose of lithium (NP03-40). On the other hand, PrP^Sc^ level, which is one of the main hallmarks of prion diseases, was not significantly changed upon lithium treatment. This lack of effect can be explained because mice were all sacrificed at the disease endpoint when PrP^Sc^ level is the highest. Moreover, PrP^Sc^ level reaches a plateau at the late disease stages. Nevertheless, NP03-treated mice presented slightly lower PrP^Sc^ levels compared with the vehicle and the lithium-gavage groups. This effect can be explained by lithium induction of the clearance of PrP^Sc^ ^13^, and also of alpha-synuclein^1^, huntingtin^11^, or amyloid peptides^40^.

Postmortem immunohistological analysis of brain tissue sections of these mice showed a significant reduction of vacuolization and astrogliosis, and a concomitant higher proportion of neurons in the hippocampus of NP03-lithium mice. These effects could explain the longer survival period of the NP03-lithium mice.

The astrogliosis reduction is in accordance with the finding that lithium decreases the activation of the transcription factor STAT3, a regulator of GFAP transcription and astrogliogenesis^34,41^. It has been shown that lithium can reduce activated STAT3 and decrease GFAP expression in rodent models of ALS, taxo-induced neuropathic pain, and lipopolysaccharide-induced inflammation^42–44^. Furthermore, lithium can stimulate neuronal survival in a rat experimental model of HD in which quinolinic acid is unilaterally infused into the striatum to mimic the HD pathology^45^. Similar effects were also recently observed in a rat model of AD upon treatment with NP03 (the same formulation used in the present study). Specifically, NP03-lithium formulation promoted GSK3β inactivation, leading to BACE1 activity inhibition via β-catenin signaling. BACE is one of the secretase involved in Aβ production, therefore its inhibition could be responsible for the reduction of Aβ deposits^24^. Multiple lines of evidence suggest that lithium mimics Wnt/β-catenin signaling through GSK3β inactivation^46,47^ and that it alleviates prion-induced synaptic damage and neuronal death partially by restoring the Wnt/β-catenin signaling pathway^48,49^. Based on these findings, we hypothesized that the higher neuron surface values observed in our lithium-treated mice could be due to activation of the Wnt-signaling pathway that is impaired in prion diseases^50^. The Wnt-signaling pathway is a highly conserved pathway that regulates cell proliferation, differentiation, survival, neuronal maturation, and synaptogenesis^50^. Wnt/β-catenin signaling impairment has been reported in AD^48,51–54^, cancer, and prion diseases^50,55^, where its impairment progressively worsens during the incubation period^50^.

Prion diseases are a group of neurodegenerative diseases characterized by multiple neuropathological hallmarks. Therefore, lithium could counteract some of the prion-induced alterations through its various effects and modulation of different signaling pathways. Several papers have described lithium as an autophagy-inducing drug^56,57^ partly on the basis of the observation that in some cell culture models of HD and PD, lithium increases autophagy through GSK3β activity inhibition and mTOR activation^1,5^. However, other studies demonstrated that lithium does not positively regulate autophagy in all pathological conditions. For instance, in cerebral ischemia and AD, lithium negatively regulates autophagy^42–44^. In our study, this could also be the case for the NP03-160 group where the number of LC3-II-positive cells was significantly diminished in the striatum. Only mice in the lithium-gavage group presented significantly increased levels of autophagy in the striatum, probably due to mTOR activation via GSK3β inactivation by high doses of lithium. On the other hand, no effect was observed in the NP03-40 group, possibly because the very low lithium dose administered did not have any effect on the autophagy-signaling pathway. Moreover, we did not observe any significant autophagy changes in the cortex in any of the three treatment groups, as previously reported in the hippocampus of a mouse model of Alexander disease^34^. The discrepancy between treatment groups and brain regions concerning lithium effects on autophagy is not surprising if we consider autophagy as a multistep cellular process that is dysregulated in the multifactorial prion diseases^58^.

In NP03-160 mice, the significant autophagy reduction in the striatum could be correlated with the lower astrogliosis levels due to inactivation of STAT3, which is also considered as an autophagy regulator^59^. In the cytoplasm, STAT3 interacts with the PKR kinase to inhibit eIF2a phosphorylation and reduce autophagy. Upon activation and nuclear translocation, STAT3 cannot interact with the PKR kinase, and this leads to eIF2A activation and autophagy induction. Therefore, lithium-induced inactivation of STAT3 in NP03-160 mice could lead to (i) decreased GFAP expression and (ii) autophagy reduction. In the other groups, where autophagy was increased while astrocyte numbers were reduced, autophagy might not be regulated by STAT3 but by other signaling pathways such as mTOR/GSK3β.

**Fig. 6:**
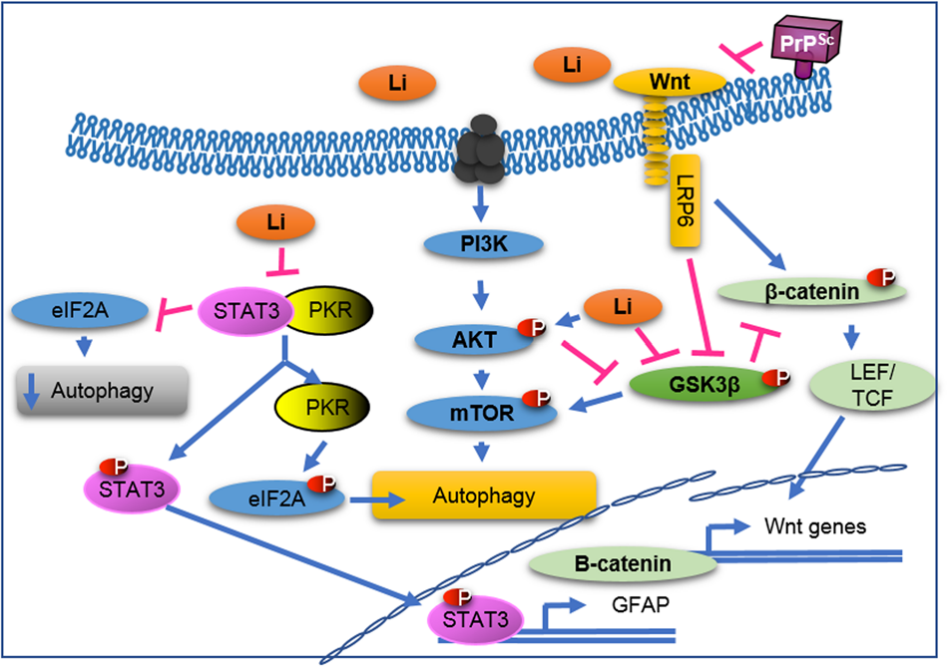
Schematic representation of the signaling pathways implicated in the improvement of the neuropathology following treatment with NP03-lithium in C57BL/6J mice inoculated with ME7 prion-infected brain homogenates. Lithium (Li) inhibits the activation of the transcription factor STAT3, a regulator of GFAP transcription and astrogliogenesis. This pathway explains the decrease of astrogliosis in mice following treatment with lithium. Lithium also restores Wnt/β-catenin signaling that is impaired in prion diseases (PrP^Sc^) through inactivation of GSK3β. This leads to transcription activation of Wnt target genes, and consequently to the limitation of neuronal loss. Lithium also increases autophagy by activation of mTOR via inactivation of GSK3β. Autophagy regulation is also affected by STAT3 activation that disrupts the interaction with PKR kinase, resulting in eIF2A activation and subsequently induction of autophagy

Figure 6 shows the multiple signaling pathways that could be modulated by lithium. These pathways are known to contribute to the amelioration of neuropathology, for instance, astrogliosis reduction through STAT3 inactivation, limitation of neuronal loss through activation of Wnt target genes, and autophagy activation by STAT3 or GSK3β.

Altogether, these results demonstrated that the Aonys^®^ NP03-lithium formulation ameliorates the neuropathology in prion models even at late stages of the disease. These results are very encouraging because the low doses of lithium delivered through the Aonys^®^ formulation were as or even more effective than the conventional high dose of lithium. Therefore, lithium delivered with the advanced NP03 formulation could be suitable for chronic treatment, while avoiding lithium-related toxicity and could help to overcome problems with toxic overdosing in human therapy.

## Electronic supplementary material

Supplemental Figure 1

Supplemental legends
