# Supplementary figures and images for: Lithium as a disease-modifying agent for prion diseases
Source: Transl Psychiatry. 2018 Aug 22;8:163. doi: 10.1038/s41398-018-0209-4 (PMC6105724; doi:10.1038/s41398-018-0209-4)

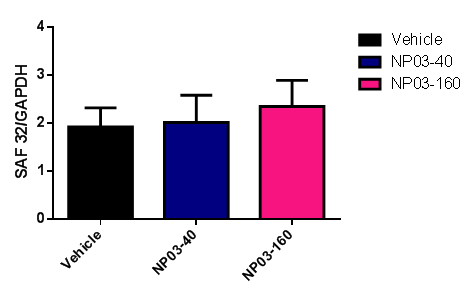

Supplement: Supplementary file 1 — Supplemental Figure 1 [file 41398_2018_209_MOESM1_ESM.tif]
