# Supplementary material for: Lithium as a disease-modifying agent for prion diseases
Source: Transl Psychiatry. 2018 Aug 22;8:163. doi: 10.1038/s41398-018-0209-4 (PMC6105724; doi:10.1038/s41398-018-0209-4)
Supplement: Supplementary file 2 — Supplemental legends [file 41398_2018_209_MOESM2_ESM.docx]

Supplementary Figure 1. PrPC Western blot analysis. PrPC levels are not affected by the treatment.
